# Heat-Assisted Pulsed Electric Field Treatment for the Inactivation of *Saccharomyces cerevisiae*: Effects of the Presence of Citral

**DOI:** 10.3389/fmicb.2019.01737

**Published:** 2019-07-31

**Authors:** Chiara Montanari, Urszula Tylewicz, Giulia Tabanelli, Annachiara Berardinelli, Pietro Rocculi, Luigi Ragni, Fausto Gardini

**Affiliations:** ^1^Interdepartmental Center for Industrial Agri-Food Research, University of Bologna, Cesena, Italy; ^2^Department of Agricultural and Food Sciences, University of Bologna, Cesena, Italy

**Keywords:** *Saccharomyces cerevisiae*, pulsed electric field, hurdle technology, citral, flow cytometry

## Abstract

Pulsed electric field (PEF) treatment is a non-thermal technology that has shown good potential for microbial inactivation. However, in many cases, it cannot be sufficient to avoid microbial proliferation, and the combination with other stabilizing technologies is needed. In the framework of the hurdle concept, several researches have been focused on the use of PEF in combination with heat and/or antimicrobials to increase its efficacy. This study investigated the inactivation effect of PEF on a strain of *Saccharomyces cerevisiae* (isolated from spoiled beverages) in a model system (growth medium). The efficacy of PEF treatment was evaluated in relation to different variables, such as electric field strength (25 and 50 kV/cm), treatment time (from 1 to 5 s), initial inoculum level (4 or 6 log cfu/ml), preheating at 50°C, medium pH (4 or 6), and addition of citral at sublethal concentration (i.e., half of minimum inhibiting concentration). The data from plate counting, modeled with the Weibull equation, showed that one of the main factors affecting yeast inactivation was the preheating of the suspension at 50°C. Indeed, higher cell load reductions were obtained with heat-assisted PEF, especially in the presence of citral. The effect of initial cell load was negligible, while pH affected yeast inactivation only without preheating, with higher death kinetics at pH 6. Flow cytometry (FCM) analysis confirmed higher mortality under these conditions. However, the occurrence of injured cells, especially in samples treated at pH 4, was observed. The ability of these cells to recover from the damages induced by treatments was affected by both citral and preheating. The synergic effects of PEF, preheating, and citral were likely due to the increase of membrane permeability (especially at pH 6), as the primary target of electroporation, which favored the solubilization of citral in the cell membrane, enhancing the efficacy of the whole process. The multi-analytical approach (traditional plate counting and FCM) allowed defining parameters to increase PEF efficacy against *S. cerevisiae*. Moreover, FCM, able to discriminate different physiological states of the yeast population, was helpful to better clarify the action mechanism and the potential recovery of cells after treatment.

## Introduction

Pulsed electric field (PEF) is considered a mild non-thermal technology for food preservation since it gives an advantage of better retention of heat sensitive food components in fruit and vegetable products (Barba et al., 2015, 2017a; González-Arenzana et al., 2015; Moussa-Ayoub et al., 2017). The electric field is delivered to the product placed between two electrodes in form of pulses of very short duration (μs), making the whole treatment very brief (Buckow et al., 2013; Jäger et al., 2014). Normally for the microbial cells, which are quite small (1–10 μm) in comparison to the plant cells (40–200 μm), a high electric field strength, in the range of 15–50 kV/cm or even up to 90 kV/cm (Liang et al., 2002), is necessary to induce cell membrane electroporation (Knorr et al., 2011; Barba et al., 2015, 2017b).

The electroporation of cell membranes results in alteration of cell membrane permeability and integrity and subsequently in the molecular exchange between cytoplasm and external medium across the lipid membrane. Thus, there is a decrease in the capacity of microbial cells to maintain homeostasis, causing the inactivation of vegetative cells at temperatures below those used in conventional thermal processing (Álvarez et al., 2006; Raso, 2016; Djukic-Vukovic et al., 2017).

PEF has been widely applied to control yeast spoilage in liquid foods. Many studies have been focused on the species *Saccharomyces cerevisiae,* known to be involved in the degradation of fruit-based beverages such as juices and soft drinks (Sperber and Doyle, 2009; da Cruz Almeida et al., 2018). Indeed, the characteristics of these products (low pH and high sugar amounts) give a competitive advantage to this species towards other microbial groups. To avoid yeast proliferation, that would cause ethanol and CO_2_ accumulation, with consequent package blowing, thermal treatments have been traditionally applied (Tabanelli et al., 2019). However, because of their negative side-effects in terms of sensorial and nutritional profiles, PEF has been proposed as a feasible non-thermal technology for beverage stabilization (Mathys et al., 2019).

Many factors (electric field strength, number of pulses, pulse width and shape, pulse frequency, and total treatment time) should be considered for the yeast inactivation. In general, an increase in the electric field strength and treatment time leads to a more pronounced inactivation effect (Elez-Martínez et al., 2004; Katiyo et al., 2017; Ou et al., 2017; Paniagua-Martínez et al., 2018). However, PEF technology application is often studied only to investigate an alternative to conventional thermal pasteurization (Aganovic et al., 2017), and in many cases, it is not possible to guarantee the quality and safety of food products with PEF treatment alone. In order to obtain a higher antimicrobial effect, the combination with other techniques such as mild heating, antimicrobials, pH reduction, etc. has been explored (Barba et al., 2017a). Aronsson and Rönner (2001) observed that the inactivation of *S. cerevisiae* cells decreased with an increase in pH. In fact, in samples with water activity close to 1 and pH 4, an inlet temperature of 30°C and PEF treatments at 25 kV/cm caused the reduction of 3.8 log units, while in samples with higher pH, cell count reduction decreased to 1.8–2.9 log units. When lower temperature was used (10°C), the inactivation even at pH 4 was only of 1.6 log units. The influence of pH and temperature on *S. cerevisiae* was also studied in different juices by Timmermans et al. (2014). The authors observed a synergistic effect between temperature and electric pulses with an inlet temperature above 36°C; hence, lower specific energy input for inactivation was required at higher temperatures. Moreover, different juice matrices resulted in a different degree of inactivation, in relation to the matrix pH. Milani et al. (2015) observed that the increase of temperature from 43 to 53°C in combination with the PEF treatment caused a higher inactivation of yeast spores in beers with different alcohol content.

Another strategy to increase PEF efficacy is its combination with plant extracts, including essential oils or their constituents, whose antimicrobial activity is widely demonstrated (Burt, 2004; Lanciotti et al., 2004; Hyldgaard et al., 2012). The increased effect of their combination is due to the fact that cell electroporation can facilitate the diffusion of these chemicals across the cell membrane, i.e., the primary target of their activity (Raybaudi-Massilia et al., 2009). Some applications in model or real systems (mainly fruit juices) are reported in the literature and have been recently reviewed by Paniagua-Martínez et al. (2018).

Citral (3,7-dimethyl-2,6-octadienal) is a mixture of two terpenoids (geranial and neral) deriving from citrus fruits and lemon-scented plants such as verbena and lemongrass. Because of its GRAS (Generally Recognized as Safe) status, citral has been widely used as food additive and flavoring, but also its strong antimicrobial activity against bacteria and fungi is recognized (Tabanelli et al., 2014; Chueca et al., 2016; OuYang et al., 2018; Sun et al., 2019).

Taking into consideration the interactions between the hurdles described above, a suitable approach can be a combination of more than two factors. In this framework, promising results could be obtained gathering PEF, mild heating, and aroma compounds, since also the synergy between heat and natural antimicrobials in increasing the yeast inactivation kinetics has been already proved (Belletti et al., 2007, 2010).

Moreover, it is known that the alteration of microbial cell membrane permeability due to the electroporation can be temporary or permanent. Indeed, mild processing can induce the presence of injured cells that needs to be taken into consideration because of their potential ability to recover during storage (Barba et al., 2017a; Schottroff et al., 2018). To address this issue, culture-independent methods such as flow cytometry (FCM) can be helpful to investigate the physiological state of cells (e.g., viability and membrane permeability) after the adopted treatments and during storage.

The aim of this work was to study the inactivation effect of PEF treatment on a strain of *S. cerevisiae* isolated from spoiled beverages. To increase its efficacy, PEF was combined with other physico-chemical factors, such as the addition of sublethal concentration of citral, the modification of pH of treatment medium (4 or 6) and the application of a preheating at 50°C.

## Materials and Methods

### Yeast Strain and Preparation of Microbial Inoculum

The strain *Saccharomyces cerevisiae* SPA belonging to the collection of the Department of Agricultural and Food Sciences (University of Bologna) was used in this study. It was isolated from spoiled soft drinks (Ndagijimana et al., 2004) and in previous trials showed resistance to thermal and non-thermal treatments (Patrignani et al., 2013; Tabanelli et al., 2019). The strain was stored at −80°C and, before the experiments, refreshed twice in Sabouraud dextrose medium (SAB; Oxoid, Basingstoke, UK) for 48 h at 28°C.

### Pulsed Electric Field Prototype, Electrical Measurements, and Treatment Conditions

The equipment used for the tests is a homemade prototype able to produce trains of pulses generated by the discharge of a high voltage capacitor (5 nF, voltage rating, 30 kV). The pulses have the typical exponential decay with a peak voltage of 10 kV and current depending on the resistive load (200 A, with a load of 50 ohm, theoretically). The pulse width also depends on the applied load (580 ns to decimate the initial voltage of 10 kV). The number of pulses during time is independent for a wide load range (22–92 ohm) with a frequency of 54 ± 2 Hz. The energy delivered to the sample is independent of its resistivity (or conductivity) being governed by the capacitor discharge law, according to which it is the product of the capacitance for the squared voltage, divided by two. The treatment duration can be adjusted, continuously, up to 10 s. The sample to be treated is inserted into a commercial electroporation cuvette (Sigma-Aldrich, Milan, Italy), with nominal capacity of 400 or 800 μl and aluminium rectangular electrodes with active surface of 1.91 cm^2^ and electrodes 2 (50 kV/cm) or 4 mm (25 kV/cm) spaced. Measurements of current and voltage to describe the discharge shape were conducted by using a current transformer (1 V/1 A) and a high voltage probe (1 V/1 kV) connected to an oscilloscope. Measurements of the conductivity of the samples contained in the 2 mm cuvette at pH 4 and 6, at 25, 50, and 60°C were carried out by means of a LCR meter, at 1 MHz (LCR-8101G, GW-Instek, Good Will Instrument Co. Ltd.). From previous measurements, the addition of citral did not show substantial modifications of the conductivity, both at 25 and 50°C.

For each condition, yeast cells in stationary phase (72 h) were resuspended (cell load 4 or 6 log cfu/ml) in fresh medium at pH 4 or 6, with addition of citral (Sigma-Aldrich, Milan, Italy) at a concentration of 300 mg/L, corresponding to 50% of minimum inhibiting concentration. This amount of citral was selected also on the basis of previous studies performed in real foods to balance the antimicrobial activity and its sensorial impact (Siroli et al., 2015). Because of its scarce water solubility, citral was previously dissolved in ethanol (Sigma-Aldrich, Milan, Italy). The final concentration of ethanol in the medium was 0.5%, in order to avoid its antimicrobial activity; moreover, a control with ethanol alone was also considered. When required by the experimental plan, the cell suspension was preheated (directly in the electroporation cuvettes) at 50°C in a heater block (MPM Instruments, Milan, Italy) and PEF treated. To define the inactivation kinetics at different conditions, samples were treated for 1, 2, 3, 4, and 5 s, then treatment was stopped to not induce a high increase of medium temperature. Temperature increase due to 5 s of treatment was from 10 to 13°C, primarily depending on sample mass in the cuvette of different volume (a large amount of energy applied with the PEF is dissipated through the aluminium electrodes, the electrical contacts, and the cuvette walls). Samples collected after each treatment were analyzed by plate counting on SAB agar. Plates were incubated at 28°C for 72 h. All trials were conducted in triplicates.

### Model Development

The Weibull equation (van Boekel, 2002) was used to fit the survivor data for each PEF treatment:

$$\log S\left(t\right)=–b{\left(t\right)}^{n}$$

where *S(t)* is the survival ratio (*N*_t_*/N*_0_), *t* is the treatment time (s), *n* and *b* are temperature dependent parameters and represent the shape factor and the rate parameter related to the location parameter, respectively (Peleg and Cole, 1998). The experiments were done in triplicate, and the data were merged to increase the number of raw data points.

### Evaluation of Yeast Cell Damage and Recovery After Pulsed Electric Field Treatments

To investigate the physiological state of *S. cerevisiae* after PEF processing, cells were analyzed immediately after a PEF treatment of 5 s (combined or not with other factors) to assess culturability (by plate counting on SAB Agar) as well as viability and membrane permeability and depolarization by flow cytometry (FCM, described in “Flow Cytometry Analysis”). Then, cells were incubated at 28°C for 6 h and periodically analyzed as reported above, to monitor the ability to recover the damage.

### Flow Cytometry Analysis

Flow cytometry (FCM) was used to monitor the physiological state of yeast cells immediately after PEF and after a 6 h recovery, to assess if cells were able to restore the damage caused by such treatments. Cell suspensions collected immediately after treatments and during recovery were analyzed with a flow cytometer Accuri C6 (BD Biosciences, Milan, Italy), using setting parameters, emission filters and thresholds according to Arioli et al. (2019). The cells were stained with SYBR-Green I (1X), propidium iodide (PI) 7.5 μM, and DiBAC_4_(3) [Bis-(1,3-Dibutylbarbituric Acid) Trimethine Oxonol] 3.0 μM as reported by Tabanelli et al. (2019), and data obtained were analyzed using the BD ACCURITM C6 software version 1.0 (BD Biosciences, Milan, Italy).

## Results and Discussion

### Electrical Measurements

**Figure 1:**
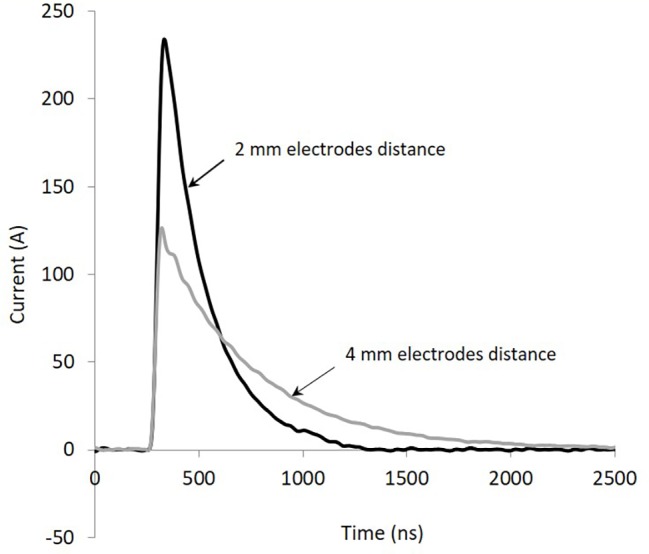
Current waveforms of the pulses with 2 and 4 mm electrode cuvette and pH 6 medium.

The waveforms of the current describing the pulses for the SAB medium, pH 6, contained in cuvettes with electrode distance of 2 and 4 mm are shown, as an example, in Figure 1. The shape is typical of the discharge of a capacitor when it is connected to a resistive load (the cuvette filled with the sample). Voltage waveforms (not shown) overlap quite well the current waveforms, being current in phase with voltage. Total energy delivered to the sample basically depends on the duration of the train of pulses (1, 2, 3, 4, or 5 s): from 13.5 to 67.5 J. Shape (duration) of each pulse varies greatly with the resistive load, according to the capacitor discharge law. Conductivity and electrode distance play a crucial role in determining the shape of the pulses. The results of the conductivity measurements carried out with SAB media at pH 4 or pH 6, for different temperatures (25, 50, and 60°C) are given in Table 1. From this table, it appears evident the influence on the conductivity (and resistance) of pH, but, above all, of the temperature. For the two media (pH 4 and pH 6), the conductivity is linearly correlated with temperature, as expected. During treatment the temperature increased (up to 13°C), the conductivity and the current increased, whereas the pulse width decreased.

**Table 1:** Conductivity of the used media. Standard deviations are reported in brackets. Measurements were conducted at 1 MHz.

Temperature (°C)	pH	Conductivity (mS/cm)
25	4	2.52 (0.01)
	6	2.30 (0.02)
50	4	4.02 (0.05)
	6	3.62 (0.01)
60	4	4.66 (0.08)
	6	4.23 (0.01)

### Effect of Different Physico-Chemical Parameters on Yeast Inactivation Kinetics

**Figure 2:**
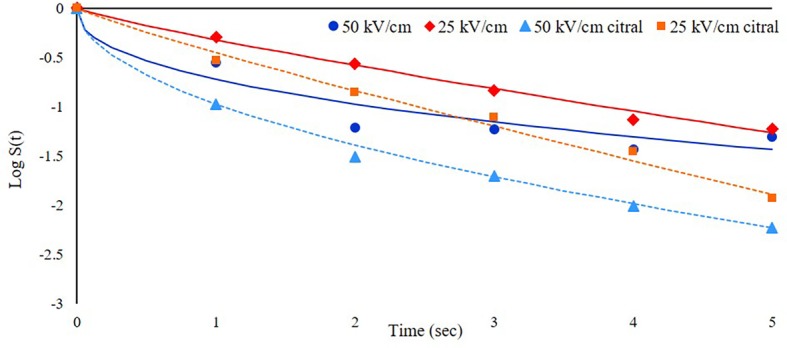
Inactivation kinetics of *S. cerevisiae* SPA during PEF treatment at 25°C and with different electric field strength (25 or 50 kV/cm) in the presence or not of citral (300 mg/L). Dots represent experimental data and lines the fitted models with Weibull equation.

The first trial was focused on the effect of different electric field intensities (25 or 50 kV/cm) on the inactivation of *S. cerevisiae* cells resuspended at a cell load of about 6 log cfu/ml in a model system (SAB medium, pH 6) with the addition of 300 mg/L of citral. The survival data were modeled with the Weibull equation (Figure 2, where dots represent experimental data as mean of three repetitions and lines the corresponding models) and showed that the inactivation kinetics increased with increasing the field intensity applied to the suspension. In particular, the inactivation rate was higher in the first second of treatments, with *b* values of 0.32 at 25 kV/cm instead of 0.75 at 50 kV/cm. The log reduction after 5 s of treatment was 1.26 and 1.68 log cfu/ml at 25 and 50 kV/cm, respectively. These results are consistent with those obtained by Martínez et al. (2016), which observed that the non-linear inactivation kinetics of a *S. cerevisiae* commercial strain were faster in the first moments of the treatment and then the survivors number decreased more slowly as treatment time increased.

Wang et al. (2016) observed a lower survival rate at higher fields (30–50 kV/cm instead of 10 kV/cm). The inactivation rate obtained with this prototype was lower than other findings reported in the literature: for example, Aronsson et al. (2005) observed a reduction higher than 6.8 log in a yeast population treated at 30 kV/cm with a pulse duration of 4 μs. This difference could rely on the different pulse duration and the medium conductivity; in this latter study, cells were treated in phosphate buffer with electric conductivity of 4.0 mS/cm and it is known that the increase of conductivity can induce lower survival of cells (Wang et al., 2016). Moreover, Aronsson et al. (2005) used a rectangular shaped pulse, which could result in a higher yeast inactivation efficiency when compared to the exponential decay pulse. In fact, Qin et al. (1994), comparing differently shaped pulse waves, observed that bipolar square-wave pulses were the most efficient in terms of microbial inactivation for commercial PEF pasteurization.

Regarding aroma compound addition, in both cases, the presence of citral increased the inactivation kinetics, with a log reduction at the end of treatment of 1.93 and 2.23 log cfu/ml at 25 and 50 kV/cm, respectively. The combination of PEF with other antimicrobials, including also essential oils or their constituents, has been already applied in both model and real systems with the aim to increase PEF efficacy (Mosqueda-Melgar et al., 2008; Ait-Ouazzou et al., 2011; Barba et al., 2017b; Paniagua-Martínez et al., 2018). However, this additive or synergic effect has not been always observed. Even if membrane permeabilization induced by PEF may facilitate the entry of antimicrobials compounds (Somolinos et al., 2007), presumably increasing the treatment efficacy, some authors reported no synergy between citral and PEF, while this positive effect has been seen when citral was combined with mild heat treatment at 53–54°C (Chueca et al., 2016).

Since the inactivation rate and the cell load reduction at the end of treatment were higher at 50 kV/cm than at 25 kV/cm, the former electric field strength was selected for the following experiments. This process parameter was indeed more promising to better investigate the phenomena involved and the potential synergistic effect of the factors then considered.

**Figure 3:**
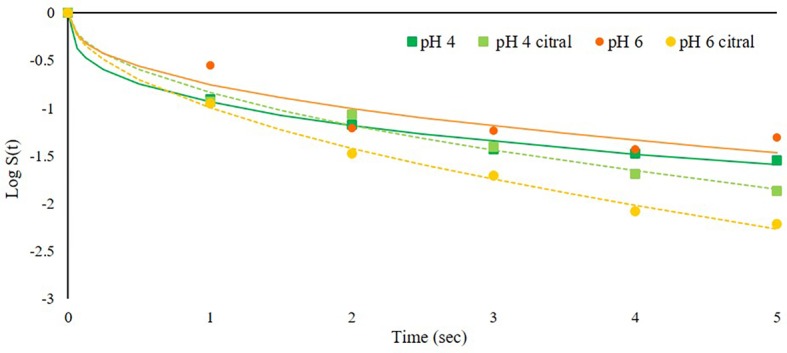
Inactivation kinetics of *S. cerevisiae* SPA during PEF treatment (50 kV/cm) at 25°C at different medium pH values (4 or 6) in the presence or not of citral (300 mg/L). Dots represent experimental data and lines the fitted models with Weibull equation.

The second trial was aimed to assess the effect of pH combined with PEF and citral, using acidified SAB medium (pH 4), while the other parameters were not modified. The results showed that only slight differences in relation to pH were observed during a 5-s treatment (Figure 3). This can be due to the fact that the optimal pH range for *S. cerevisiae* growth can vary from pH 4 to 6 depending on the strain or environmental parameters such as oxygen and temperature (Fakruddin et al., 2012). Also, Aronsson and Rönner (2001) observed low inactivation rate at pH near 5, while PEF efficacy against yeasts increased moving toward higher pH. The strain used in our study was isolated from spoiled beverages (soft drinks with pH values of 3–3.2), and it was therefore adapted to low pH environment (Ndagijimana et al., 2004). The pH values tested in the present trial likely did not strongly affect the yeast response to PEF treatment when no other factors were modified, i.e., without antimicrobials. However, differences related to pH were observed when citral was added to the treatment medium: indeed, the inactivation kinetics slightly increased at higher pH 6. This was an unexpected behavior, since the literature generally reports that essential oils and aroma compounds are more effective at acidic pH (Burt, 2004). However, our findings agree with those by Onawunmi (1989) for citral, which at acidic pH decreased its antimicrobial activity against bacteria and fungi. The reason relies on the low chemical stability of citral under acidic aqueous conditions, in which it can easily degrade to a variety of by-products (Ueno et al., 2004).

The effect of citral and medium pH was assessed also at a lower inoculum level (4 log cfu/ml instead of 6). No differences in relation to initial cell concentrations were observed (data not shown). The literature regarding the effect of inoculum level on PEF inactivation is contradictory. Some authors reported that, in the range 3–8 log cfu/ml, the rate of *S. cerevisiae* inactivation increases with decreasing the initial cell concentration (Donsì et al., 2007) while, according to other researches, microbial inactivation by PEF treatment is unaffected by the initial inoculation level (Barbosa-Cánovas et al., 1999; Álvarez et al., 2000). Since in our trials the inoculum level did not affect cell inactivation, the following experiments were performed using only the higher inoculum level, to allow also FCM analyses.

### Effect of Preheating Combined With pH and Citral on Pulsed Electric Field Efficacy Against *S. cerevisiae*

**Figure 4:**
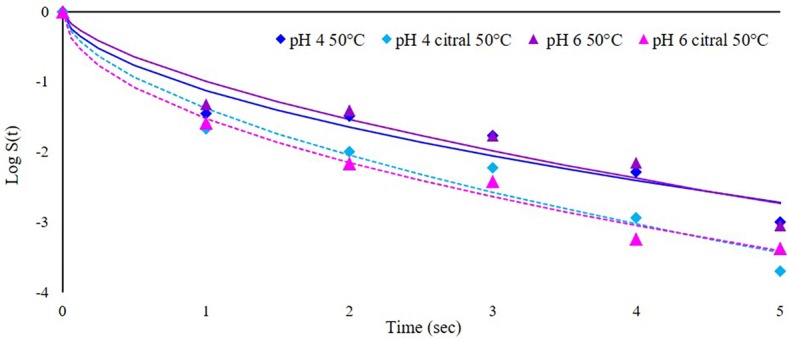
Inactivation kinetics of *S. cerevisiae* SPA during heat-assisted (50°C) PEF treatment (50 kV/cm) at different medium pH values (4 or 6) in the presence or not of citral (300 mg/L). Dots represent experimental data and lines the fitted models with Weibull equation.

This part of the study was focused on the application of heat-assisted PEF to increase its efficacy, preheating the cell suspension at 50°C immediately before treatment. In this condition, treatment was stopped after 5 s because the temperature increase was about 13°C, and longer PEF application would have resulted in additional thermal treatment. It is worth mentioning that the effect of preheating temperature alone is negligible: preliminary trials showed that at 50°C the time needed to inactivate 5 log cycle (T_5log_) of *S. cerevisiae* SPA was higher than 500 min. In addition, also at the highest temperature reached at the end of heat-assisted PEF treatment (63°C), the T_5log_ was higher than 20 min (data not shown). Survivor data of plate counting were modeled with the Weibull equation since the inactivation kinetics were not linear (Peleg and Cole, 1998; van Boekel, 2002). The results are reported in Figure 4, where dots represent experimental data (mean of three repetitions) and lines the corresponding models. The synergic effect of heat and PEF is evident from the inactivation curves, with a log reduction at the end of the treatment (5 s) of about 3 log cfu/ml, independently of medium pH. The addition of citral at sublethal concentration further increased this rate (of about 0.5 log) but even in this case, the effect of pH was negligible. To facilitate the comparison between the different experiments and the effects of the combined hurdles (PEF, pH, citral, preheating), the parameters estimated by the Weibull equation (*b* and *n*), together with the diagnostics of fitting (*R*, *F*, *p*, and RMSE) and the log reduction (cfu/ml) predicted by the models after 5 s of treatment (log red_5sec_) are summarized in Table 2.

**Table 2:** Parameters estimated by Weibull equation (*b* and *n*), with the diagnostics of fitting (R, *F*, *p*, and RMSE) for the inactivation curves of *S. cerevisiae* SPA in different conditions. The log reduction (cfu/ml) predicted by the models after 5 s of treatment (Log red_5sec_) are also reported.

Conditions		*b*	*n*	*R*	*F*	*p*	RMSE	Log red_5sec_
PEF treatment at room temperature (25°C)	pH 4	0.938	0.330	0.997	1766.74	0.0000	0.04	1.59
	pH 4 citral	0.839	0.491	0.981	648.14	0.0001	0.06	1.85
	pH 6	0.752	0.415	0.961	115.84	0.0003	0.14	1.47
	pH 6 citral	0.996	0.510	0.994	1805.96	0.0000	0.05	2.26
Heat-assisted (50°C) PEF treatment	pH 4 50°C	1.126	0.547	0.969	140.77	0.0002	0.22	2.72
	pH 4 citral 50°C	1.387	0.564	0.982	242.98	0.0001	0.22	3.44
	pH 6 50°C	0.993	0.630	0.969	130.19	0.0002	0.23	2.74
	pH 6 citral 50°C	1.529	0.498	0.994	759.88	0.0000	0.12	3.41

In all the tested conditions, the estimates of *n* were always lower than 1, indicating the presence of a “tail,” likely due to the presence of a subpopulation endowed with higher resistance or the ability of some cells to adapt to treatment applied. Similar results are reported in the literature (Donsì et al., 2007), even if some authors observed a different behavior, with inactivation kinetics characterized by a shoulder, indicating that during treatment the cells accumulated damage and became weaker with increasing temperature (Timmermans et al., 2014). The same authors, however, postulated that it is very difficult to compare these contradictory results since they can strongly vary according to PEF equipment (treatment chamber, setting parameters, etc.).

The *b* values generally increased in samples preheated at 50°C, with higher values in the presence of citral, and indicated a more rapid inactivation rate. Some previous studies have described the effect of processing temperature on the inactivation of *S. cerevisiae*. For instance, Aronsson and Rönner (2001) and Timmermans et al. (2014) observed a significant increase of the inactivation rate of this yeast species when the inlet temperature increased above 30–36°C before PEF treatment. The synergy between these factors is due to the alteration of the fluidity of cell membrane, caused by higher temperature, that enhances electroporation (Ou et al., 2017). In addition, the higher temperature strengthens the antimicrobial activity of citral since, increasing vapor pressure, enhances its solubilization into the cell membrane (Hyldgaard et al., 2012).

Therefore, even if the single hurdles at this extent are insufficient to inactivate microorganisms, they can act synergistically and result in a lethal treatment. Regarding the further addition of antimicrobials in such conditions, Liang et al. (2002) achieved a reduction of 4.4 log cfu/ml combining PEF treatment (87 kV/cm), mild heat (52°C), and a mixture of lysozyme and nisin. While the double combinations PEF/heating and PEF/natural antimicrobials have been investigated in yeasts and bacteria (Martín-Belloso and Sobrino-López, 2011; Paniagua-Martínez et al., 2018), to our knowledge, no researches focused on the combination of these three factors against yeasts. An interesting study was performed on spoiling microorganisms including also yeasts by Chueca et al. (2016), who investigated the interactive effects of 200 mg/L of citral with PEF (25 kV/cm) or mild heating (54°C for 10 min). These authors observed a synergy only between thermal treatment and citral, while PEF did not act synergistically with citral, contrary to the results of the present study. This could be due to the higher electric field strength applied in our trial (50 kV/cm) that could have also facilitated the entry of citral, increasing its antimicrobial activity.

### Evaluation of Yeast Cell Damage Induced by Pulsed Electric Field Treatments and Potential Recovery

After assessing the inactivation kinetics in the different conditions, the research was focused on the physiological state of yeast cells immediately after treatment and after storage. In fact, recent studies on the survival state of *S. cerevisiae* exposed to PEF have demonstrated the presence of sublethally injured cells (Paniagua-Martínez et al., 2018; Schottroff et al., 2018), whose extent depends on treatment conditions (electric field strength, pulse shape, medium, etc.). In general, the literature reports that the lethality of PEF treatment can be increased using electric field strengths above 10 kV/cm. However, Somolinos et al. (2007, 2008) clearly demonstrated the occurrence of sublethally injured yeast cells also at stronger treatments (12–20 kV/cm) and their ability to recover. Similar findings were reported for *S. cerevisiae* cells exposed to chemical or thermal stresses (Davey and Hexley, 2011). To better investigate the physiological response of the target strain, a FCM analysis of the yeast population immediately after different PEF treatments (5 s) and during recovery was performed.

**Figure 5:**
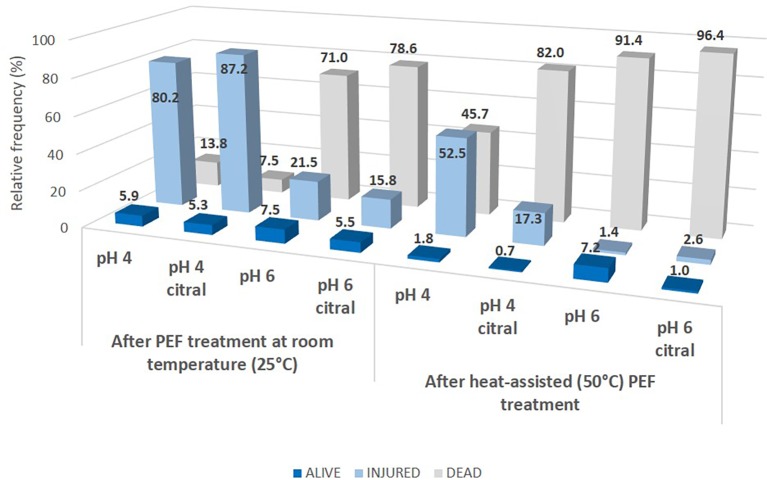
Distribution of alive, injured and dead cells of *S. cerevisiae* SPA after 5 s of PEF treatments in different conditions. Data are reported as the relative frequency of the total population obtained with dual staining (SYBR-Green I and PI) FCM analysis.

The results of the dual staining procedure are reported in Figure 5, where the percentages of alive/injured/dead cells immediately after treatments are shown as relative frequencies. When the treatments were performed without heating, at pH 6, most of the population was recognized as dead (about 70%), i.e., stained only with PI, and this percentage slightly increased with citral addition (78%). On the contrary, at pH 4, only a small percentage of cells resulted dead (14% in the control, 8% in the presence of citral), and the percentage of injured cells reached 80% of the total population in the control and 87% when citral was added at the suspension. Our results are different from those obtained through FCM by Ferrario et al. (2014) and Ferrario and Guerrero (2017), where a direct transition alive/dead was observed in *S. cerevisiae* cells treated with pulsed light and ultrasound in apple juices characterized by low pH (about 3.5) or peptone water at pH 5.6. However, Somolinos et al. (2007, 2008) reported no differences in the extent of sublethal injury (assessed by plate counting on selective agar media) in relation to pH values (4 or 7). A similar FCM approach, but with Rhodamine 123-propidium iodide dual staining, was performed by Zhao et al. (2014) to assess the damage induced by PEF treatment (20 kV/cm, square-wave pulsed) of yeast cells resuspended in phosphate saline buffer (pH 7.2, conductivity of 2 mS/cm). They observed more than 90% of injured cells that were not able to recover in a selective medium due to the damaged osmoregulatory functions of the cytoplasmic membrane.

When the cell suspensions were preheated before PEF (Figure 5), the lethality of treatment increased, with a higher percentage of dead cells, especially at pH 6, where these values reached 96 and 91% with or without citral, respectively. Similar behavior was observed at pH 4 only in the presence of citral (82% of population recognized as dead) while without the aroma compound almost half of the population (46%) was still injured. Noteworthy, these data differed from those of plate counting since only for samples preheated and then PEF-treated in the presence of citral, a complete loss of culturability was observed, with values below the detection limit (<1 log cfu/ml, data not shown). These findings are reported also in other studies that postulate that loss of culturability (i.e., inability to grow on plates) cannot be necessarily correlated to the cell death or injury, since plate counts can underestimate or overestimate the true viability of a population (Davey, 2011; Ambriz-Aviña et al., 2014). For example, Ferrario et al. (2014) described the occurrence of a subpopulation of PEF treated *S. cerevisiae* cells unable to grow on plates but still with metabolic activity.

**Figure 6:**
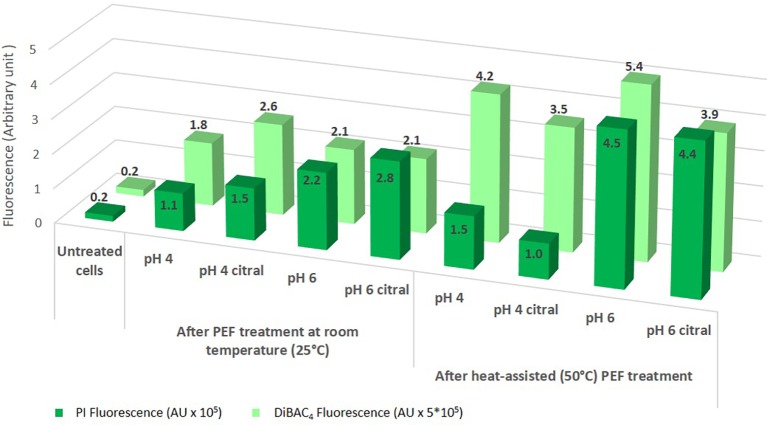
Membrane permeability (mean PI fluorescence as an arbitrary unit, AU) and membrane depolarization [mean DiBAC_4_(3) fluorescence as an arbitrary unit, AU] of stained cells of *S. cerevisiae* SPA after 5 s of PEF treatments in different conditions.

The different physiological responses in relation to medium pH and preheating can be related to differences in membrane permeability and potential (Figure 6). Indeed, when treatment was performed at room temperature, samples at pH 6, characterized by high percentage of dead cells, showed enhanced PI fluorescence with respect to cells treated at pH 4, indicating an alteration of membrane integrity that increases its permeability. This trend was confirmed also when the suspension was preheated, even if with values much higher for samples at pH 6. It is important to point out that in heat-assisted PEF treatment, the permeabilization of yeast cell membrane was not correlated to culturability since its loss was dependent on citral addition rather than pH values of the treatment medium. Aronsson et al. (2005) also reported that cells of *S. cerevisiae* whose cell membranes were permeabilized did not lose their ability to multiply.

The data regarding cell membrane depolarization showed that it increased as an effect of PEF treatment and, even more, of heat-assisted PEF treatment (Figure 6). It is worth mentioning that the entry of DiBAC_4_(3), as a measure of membrane depolarization, requires a lower damage than the entry of PI (increased membrane permeability) (Davey and Hexley, 2011); therefore, modification in membrane potential can be the first evident effect of treatments but it is not necessarily correlated to significant damage of cells.

After treatments, cells were incubated at 28°C to monitor the possible recovery within 6 h (Table 3), since it is known that under certain circumstances (presence of nutrients, proper pH and temperature), microorganism might be able to reseal the membrane pores and therefore regain viability (Schottroff et al., 2018). Regarding yeast population treated at room temperature, a general slight increase of dead cells was observed, with the exception of the samples treated without citral, where part of the population was able to restore its viability (about 10 and 18% after 6 h, instead of 6 and 8% immediately after treatment at pH 4 or 6, respectively). While Somolinos et al. (2008) reported that the recovery of treated yeast cells was favored by low pH, in our study, this ability seemed to be more related to the citral presence rather than pH value of the medium. When PEF was combined with preheating, during the 6 h of recovery in all the conditions the percentage of dead cells increased, reaching values higher than 90 and 95% at pH 4 and 6, respectively. Based on these results, the conditions adopted seem to be able to inhibit a potential recovery of injured cells, in particular when citral was added and, even more, when preheating was performed.

**Table 3:** Flow cytometry analysis of *S. cerevisiae* SPA during recovery after 5 s of PEF treatments in different conditions.Data are reported as distribution of alive, injured and dead cells as relative frequency (% of the total population) obtained with dual staining (SYBR-Green I and PI) and mean PI and DiBAC_4_(3) fluorescence of stained population (arbitrary unit, AU). Values are the average of three independent experiments; the standard deviation was always less than 5%.

Conditions			% Population			PI fluorescence	DiBAC_4_ fluorescence
			Alive	Injured	Dead	AU × 10^5^	AU × 5 × 10^5^
After 3 h of recovery	PEF treatment at room temperature (25°C)	pH 4	5.19	70.02	24.80	1.19	3.09
		pH 4 citral	4.71	69.70	25.59	1.33	3.03
		pH 6	6.56	11.83	81.61	2.61	2.30
		pH 6 citral	4.77	10.37	84.85	2.81	2.04
	Heat-assisted (50°C) PEF treatment	pH 4	1.52	3.22	95.27	1.20	3.64
		pH 4 citral	0.41	12.70	86.89	0.90	2.68
		pH 6	2.31	1.05	96.65	4.16	4.95
		pH 6 citral	0.78	1.55	97.67	4.16	3.18
After 6 h of recovery	PEF treatment at room temperature (25°C)	pH 4	9.75	79.78	10.47	1.12	2.83
		pH 4 citral	4.60	77.40	18.00	1.15	2.74
		pH 6	18.37	5.90	75.73	2.01	1.76
		pH 6 citral	4.38	4.29	91.32	2.23	1.34
	Heat-assisted (50°C) PEF treatment	pH 4	1.27	5.26	93.47	1.37	3.64
		pH 4 citral	0.43	5.43	94.13	0.97	3.21
		pH 6	2.66	0.67	96.67	3.86	4.46
		pH 6 citral	0.91	1.13	97.96	3.71	3.70

The PI fluorescence of treated yeast population slightly decreased during incubation, likely because part of the cells was able to restore the integrity of cell membrane after 6 h, especially in samples treated at pH 6 without preheating. Indeed, the reversible permeabilization of *S. cerevisiae* cells subjected to thermal or electric stresses has been already reported in the literature (Aronsson et al., 2005; Davey and Hexley, 2011; Ou et al., 2017).

## Conclusions

PEF is generally recognized as a promising non-thermal technology, but to achieve a proper inactivation rate of *S. cerevisiae*, its combination with other factors is needed.

In this study, we demonstrated a synergic effect of PEF with citral (added at concentration unable to affect cell viability, i.e., half of minimum inhibiting concentration) and increased treatment temperature (50°C). Inactivation kinetics showed a higher efficacy of PEF combined with citral at pH 6. These results are in agreement with FCM analysis, which evidenced a higher lethality in those conditions. However, it is important to take into consideration that these treatments, especially when performed in the medium at pH 4, induced the occurrence of an injured population, which in some cases was able to revert to a viable status during 6 h of recovery at the optimal growth temperature. The presence of citral inhibited this ability, suggesting that this aroma compound could be used to increase not only the efficacy of treatment but also the stability of treated samples during storage. Moreover, also the application of mild heating before PEF strongly affected the potential recovery of yeast cells.

FCM allowed discriminating different physiological states of yeast population: this approach is therefore helpful to better clarify the action mechanism of PEF, known to be exerted primarily at cell membrane level, but still not completely understood.

Since this research was performed in a model system, we cannot exclude that the proportion of cells able to repair the damages would be higher in more complex systems (such as fruit juice and beverages), where yeasts represent the main spoilage microflora. Further researches are needed to optimize process parameters in order to minimize this risk and, when combined with other hurdles, to assess the contribution of each factor in increasing the inactivation rate. In addition, the quality of the final product has to be assessed in terms of sensorial impact of the stabilizing strategy, in particular the use of citral, that in some conditions can significantly affect aroma profile and therefore needs to be adjusted in relation to the specific matrix.

The prototype-to-industrial scaling-up should not present difficult engineering problems. However, it will be necessary to consider the constraints of the required flow rate and the residence time between the electrodes of the fluid to be microbiologically inactivated.

## Data Availability

All datasets generated for this study are included in the manuscript and/or the supplementary files.

## Author Contributions

CM set up the experimental plan, performed flow cytometry analyses, elaborated the data, and wrote part of the manuscript. UT set up the experimental plan and wrote part of the manuscript. GT performed microbiological analyses, elaborated the data, and wrote part of the manuscript. AB elaborated part of the data and revised the manuscript. PR set up the experimental plan and revised the manuscripts. LR designed and realized the PEF prototype used in the study, performed PEF treatments, and wrote part of the manuscript. FG set up the experimental plan, elaborated the data, and wrote part of the manuscript.

### Conflict of Interest Statement

The authors declare that the research was conducted in the absence of any commercial or financial relationships that could be construed as a potential conflict of interest.
